# Protective Effects of Chicken Egg Yolk Immunoglobulins (IgYs) against *Vibrio vulnificus* Infections

**DOI:** 10.1155/2021/6678513

**Published:** 2021-01-06

**Authors:** Ruizhao Cai, Ning Liu, Penghao Guo, Kang Liao, Mengzhi Li, Junyou Zhu, Shouyi Chen, Lei Chen, Bin Shu, Shaohai Qi

**Affiliations:** ^1^Department of Burns, The First Affiliated Hospital of Sun Yat-sen University, Guangzhou, 510080 Guangdong Province, China; ^2^Guangdong Engineering & Technology Research Center for Precise Diagnosis and Treatment of Burns and Wounds, Guangzhou, 510080 Guangdong Province, China; ^3^Department of Clinical Laboratory, The First Affiliated Hospital of Sun Yat-sen University, Guangzhou, 510080 Guangdong Province, China; ^4^Guangzhou Center for Disease Control and Prevention, Guangzhou 510080, Guangdong Province, China

## Abstract

*Vibrio* (*V.) vulnificus* infection is a rare disease whose death rates exceed 50% despite aggressive antibiotic treatment and surgical debridement. The aim of this study was to assess the ability of specific anti-*V. vulnificus* immunoglobulins Y (IgYs) for preventing and treating *V. vulnificus* infections. IgYs were produced by immunizing egg laying hens with inactivated whole cell bacteria. Peritoneal cytokines, blood's bacterial load, and survival curves were obtained from both prophylactic and therapeutic mouse models. The results showed that the specific IgYs (i) inhibited the growth of *V. vulnificus* in vitro, (ii) dramatically reduced the inflammatory response and blood's bacterial load, and (iii) improved the survival rate of *V. vulnificus*-infected mice. These results prove that anti-*V. vulnificus* IgYs can be markedly effective means for the prophylaxis and the therapy of *V. vulnificus* infections.

## 1. Introduction

*Vibrio* (*V.) vulnificus* is a halophilic Gram-negative pathogen that is naturally present in estuarine waters, contaminated oysters, and other shellfish [1, 2]. *V. vulnificus* infection may progress to severe skin lesions and septicemia in people with predisposing conditions, including liver diseases, hereditary hemochromatosis, and a compromised immune system [2, 3]. Infection by this agent is the prominent cause of seafood-related deaths. Notwithstanding aggressive interventions like surgical debridement and antibiotic treatment, *V. vulnificus* infection-caused septicemia has a mortality rate equal to or higher than 50%. And with no antibiotic therapy, this figure could raise up to 100% [3]. During the past decades, the annual incidence of *V. vulnificus* infections has been increasing in parallel with global warming [4]. However, the methods and effects of the current treatments against *V. vulnificus* infections are still inadequate. Moreover, although *V. vulnificus* is usually vulnerable to most antibiotics, several studies have reported that it could resist multiple antibiotics because of the ongoing misuse of antibiotics in aquaculture farming [5].

Antibody therapy is potentially one of the most important methods that can replace antibiotic treatment [6]. Compared with antibiotics, antibody therapy does not present resistance problems; this makes mass prophylactic medications workable. Egg yolk immunoglobulins Y (IgYs) are the major antibodies found in chicken egg yolk. Phylogenetic evidence shows that IgYs may be the evolutionary ancestors of both mammalian IgGs and IgEs; notably, IgYs exhibit functional similarities to IgGs [7]. Since eggs are a quite common staple of human diet that is tolerated by the human immune system, it is widely accepted that IgYs do not induce any human mucosal immunity [8].

As compared with IgGs, the noninvasive, low-cost, and simple collection method from egg yolk makes IgYs into favorable therapeutic agents capable of controlling infectious diseases [9]. In fact, IgYs immunotherapy, widely used in livestock and aquaculture, has given excellent results. Because of them, IgY treatment of clinical gastrointestinal and oral pathogens infections has also attracted attention [9, 10].

Therefore, the aim of the present study was to explore the capability of IgYs obtained via immunization against inactivated whole-cell *V. vulnificus* to prevent and treat *V. vulnificus* infection in the respective mouse model.

## 2. Materials and Methods

### 2.1. Bacterial Strains and Cultures

The Laboratory Department of the First Affiliated Hospital of Sun Yat-sen University kindly provided the *V. vulnificus* strain (No.182487342) we used for this work. The strain was isolated from the blood of an infected patient and cultured in thiosulfate citrate bile salt sucrose agar, blood agar plates, and 3% sodium chloride alkaline peptone broth (all from Hopebio, China).

### 2.2. Ethic Statement for Animal Use

Male CD1 mice, aged 6 to 8 weeks, were bought from Liaoning Changsheng Biotechnology Co., Ltd. (Liaoning, China). The 20-week-old, roman powder laying hens were purchased from Zhengda Egg Industry Co., Ltd. (Sichuan, China). All animal experiments were conducted according to protocols approved by the Animal Ethics Committee of Sun Yat-sen University. The design of all the mouse experiments aimed at minimizing the animals' numbers. All efforts were made to reduce to a minimum the animals suffering and distress.

### 2.3. Immunization and Collection of IgY Antibodies

*V. vulnificus* bacteria were killed by incubating them with 0.4% formaldehyde at 37°C for 24 h, next washed with phosphate-buffered saline (PBS), and stored frozen until used. A 1 : 1 mixture of complete Freund's adjuvant with 1.8 ml of 1 × 10^9^ CFU/ml bacteria was injected into the hens' breast muscle. Next, to strengthen immunization at weeks 4, 8, and 12, the hens were injected a 1 : 1 mixture of 1.8 ml of 1 × 10^9^ bacterial CFUs with incomplete Freund's adjuvant.

Eggs were collected until the detected IgYs' titers were over 1 × 10^4^. The separated egg yolks were mixed with distilled water (pH 5) at 4°C overnight. Next, the suspensions were centrifuged at 10,000 rpm for 10 min. The obtained supernatants were salted out by adding 30% ammonium sulfate. Following centrifugation, the crude extracts were dissolved in PBS and finally passed through a ultrafiltration cartridge tube type 100 KD UF to concentrate the proteins.

### 2.4. Assessment of IgY Titers and of Bacterial Growth Inhibition

An enzyme-linked immunosorbent assay (ELISA) served to figure out the level of IgYs against whole-cell *V. vulnificus*. Briefly, 100 *μ*l of inactivated bacteria was incubated overnight at 4°C. The coated plates were washed three times with 0.05% Tween-PBS and blocked with 5% bovine serum albumin. Next, the plates were incubated with serial dilutions of IgYs and washed again with Tween-PBS. This was followed by an incubation with 100 *μ*l of 1 : 10,000-diluted HRP-conjugated rabbit anti-IgY antibody. Finally, after adding the TMB substrate, color development was stopped with 2 M H_2_SO_4_. The absorbance was measured at 450 nm in a microplate reader (PEIOU, China).

To perform the growth inhibition assays, the bacterial density was adjusted to 10^5^ CFU/ml in 3% sodium chloride alkaline peptone broth. Next, the specific and nonspecific IgYs were added to the bacterial broth, making the final concentrations of the specific IgYs equal to 2, 1, and 0.5 mg/ml and that of the nonspecific IgYs equal to 2 mg/ml. An alike volume of plain PBS was added to the control group. Finally, the optical density (OD) was read at 600 nm.

### 2.5. Mouse Experiments

To assess the prophylactic and therapeutic efficacy of the specific anti-*V. vulnificus* IgYs, 8-week-old CD1 mice were intraperitoneally injected with 2 × 10^6^ bacterial CFUs (~23.3 LD_50_) at a given time point before or after the administration of a single dose of IgYs. The mice from the experimental groups were given the specific IgYs produced by hens immunized with inactivated whole-cell *V. vulnificus*. The mice from the negative control group received nonspecific IgYs produced by hens with no earlier immunization, and the mice from the blank control group received plain PBS with no IgYs.

### 2.6. ELISA Assessment of Mice Peritoneal Lavage Cytokines, Quantitation of Blood Bacterial Load, and Mice Survival Curves

Peritoneal lavages with 0.5 ml saline were performed 5 hours after *V. vulnificus* infection. TNF-*α* levels in lavage fluids were determined via ELISA. Meanwhile, whole blood samples were collected from the orbits of infected mice to assess the numbers of blood stream live bacteria via the plating method. Finally, the survival curves of *V. vulnificus* infected mice were evaluated.

### 2.7. Statistical Analysis

All data were graphed and analyzed using GraphPad Prism version 8 (San Diego, CA). The levels of statistical significance for ELISA assays, bacterial growth inhibition assays, TNF-*α* levels, and blood bacterial load experiments were found by using a *t*-test for pairwise comparisons. Kaplan-Meier survival curves were analyzed using the log-rank test. All the results were expressed as means ± standard deviations (SDs). *p* values<0.05 were considered significant.

## 3. Results

### 3.1. Anti-*V. vulnificus* IgY Titers

**Figure 1:**
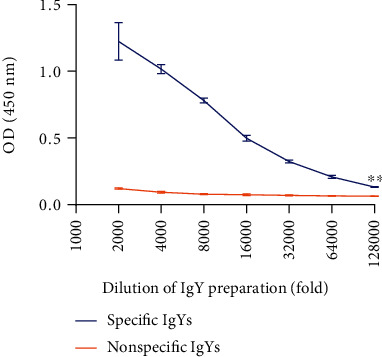
The titers of specific anti-*V. vulnificus* IgYs at different dilutions as assayed via an indirect ELISA ^∗∗^*p* < 0.01.

We used an indirect ELISA assay to figure out the anti-*V. vulnificus* IgY titers. The hens immunized with inactivated whole-cell *V. vulnificus* had significantly higher IgY titers than control hens had. Moreover, the IgY titers were correspondingly reduced by gradual increases in *V. vulnificus* antigen dilution. This showed that the antigens had successfully stimulated the IgY antibodies production in the hens (Figure 1). The specific IgY titers reached the highest level by 16 weeks after the first immunization and persisted for 4 weeks after the last (data not shown).

### 3.2. Bacterial Growth Inhibition Assay

**Figure 2:**
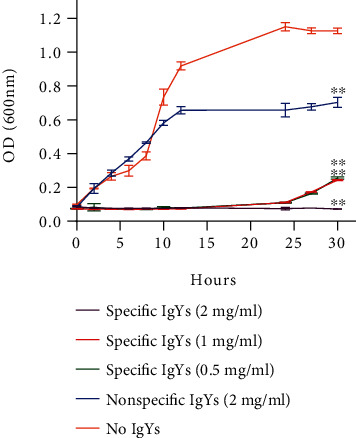
The growth inhibitory effects of specific anti-*V. vulnificus* IgYs as elicited with different antibody concentrations ^∗∗^*p* < 0.01.

As compared to the no IgY-added group, a significant reduction of *V. vulnificus* growth became manifest 30 hours after the incubation with specific IgYs in doses from 0.5 to 2 mg/ml. Increasing the specific IgYs concentration brought about more intense inhibitory effects (Figure 2). Weaker inhibitory effects were seen also after adding the nonspecific IgYs. Therefore, the specific IgYs did more intensely inhibit the growth of *V. vulnificus* bacteria in vitro (Figure 2).

### 3.3. Specific Anti-*V. vulnificus* IgYs Reduced the Inflammatory Response and the Blood Bacterial Load

**Figure 3:**
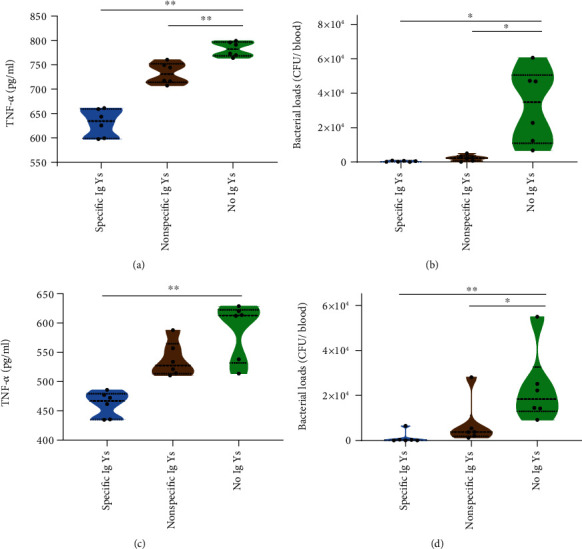
The effects of IgYs against *V. vulnificus* infection on intraperitoneal lavage fluid TNF-*α* levels and on blood bacterial loads in both the prophylactic (a, b) and therapeutic (c, d) mouse models (*n* = 6) ^∗^*p* < .0.5, ^∗∗^*p* < 0.01.

In both the prophylactic and therapeutic mouse models, the levels of the peritoneal lavage proinflammatory cytokine TNF-*α* were significantly lower in the specific IgY-treated group than in the no IgY- and the nonspecific IgY-treated groups. These results showed that the administration of the specific IgYs could mitigate the inflammatory response to *V. vulnificus* infection (Figures 3(a) and 3(c)).

Moreover, since *V. vulnificus* infection causes septicemia, we evaluated the effect of specific IgYs on the blood bacterial load to better clarify the protection mechanism. Interestingly, after 5 hours of *V. vulnificus* infection, the bacterial levels in the blood of control group mice were much higher than those in the IgY antibodies group. These findings showed that the prevention or treatment with specific anti-*V. vulnificus* IgYs could effectively protect against the lethal infection by reducing the bacterial load in the blood stream (Figures 3(b) and 3(d)).

### 3.4. Effect of IgYs on the Survival of *V. vulnificus* Infection

**Figure 4:**
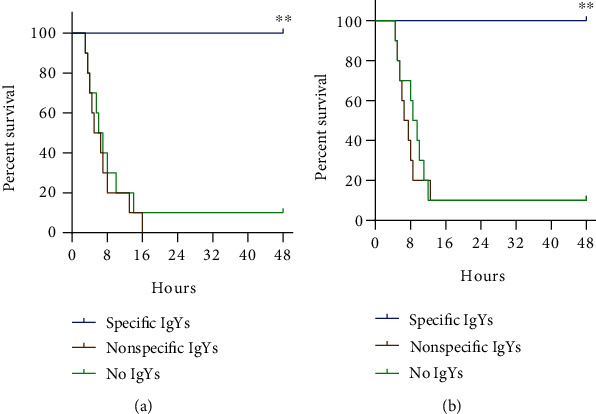
The survival protective effects of anti-*V. vulnificus* IgYs in both the prophylaxis (a) and therapeutic (b) animal models (for each group, *n* = 10). ^∗∗^*p* < 0.01.

To evaluate the prophylactic effectiveness of IgYs on the survival following *V. vulnificus* infection, CD1 mice were injected with 2 × 10^6^ bacterial CFUs (~23.3 LD_50_) 2 hours after the intraperitoneal administration of a single dose of IgYs (2 mg/ml in 0.5 ml) or of plain PBS (0.5 ml). The specific IgYs elicited a 100% protection against the death from *V. vulnificus* infection, as compared to the 10% survival of the control (no IgYs) group (*n* = 10, *p* < 0.0001) (Figure 4(a)).

We further evaluated the therapeutic effectiveness of the specific IgYs. After infection through the intraperitoneal route, CD1 mice were treated with a single dose of IgYs (2 mg/ml in 0.5 ml) or PBS (0.5 ml) 1 h. The survival of the specific anti-*V. vulnificus* IgY-injected mice was 100%. By contrast, only 10% of the control group mice survived (*n* = 10, *p* < 0.0001) (Figure 4(b)). These results showed that the specific IgYs elicited sufficient prophylactic and therapeutic effects against *V. vulnificus* infection.

## 4. Discussion

To our knowledge, this is the first proof that the specific IgYs produced by laying hens immunized with inactivated whole *V. vulnificus* exert excellent prophylactic and therapeutic effects on this bacterial infection by significantly mitigating the inflammatory response and microbial load in the blood, and by increasing the survival rate of the infected mice.

*V. vulnificus* infection is one of the main causes of seafood-related deaths. The infection occurs through accidental skin cuts while handling contaminated aquatic products, or by eating raw seafoods, such as lobster [11]. The onset of *V. vulnificus* infection is quick and it progresses fast. Notwithstanding an aggressive antibiotic therapy and surgical interventions, the fatality rate stays high [4]. Early prevention is the key to improve the prognosis. However, large-scale prophylactic antibiotic treatment would inevitably bring about serious antibiotic resistance problems. Nowadays, a resistance to multiple antibiotics has been reported for *V. vulnificus* strains isolated worldwide due to the use of antibiotics by the aquaculture farming [5]. Therefore, it is important to find other more effective methods for prevention and treatment. Among them, antibody therapy is one of the most promising means of antibiotic substitution therapy [7, 10]. Earlier studies have shown that using specific IgG serum to prevent and treat *V. vulnificus* infection gave excellent results [12, 13]. However, IgGs' preparation is complex and expensive, which makes achieving industrial production difficult [9].

Unlike IgGs that are extracted from mammalian serum, IgYs are obtained from the egg yolk by a noninvasive procedure. Each immunized hen functions as a small manufacturing plant, producing a steady stream of targeted antibodies and transferring them into the egg yolk. The cumulative amounts of antibodies produced by each hen are much higher than those extracted from the serum of a mammal. This makes the large-scale production of IgYs workable at a cost that is much lower than that of IgGs [6, 9]. Nowadays, IgYs have been widely used in animal husbandry and aquaculture, with excellent therapeutic results. In human health, IgYs have been used in mouthwash and energy drink supplements and to treat intestinal diseases such as *Helicobacter pylori* infections. Although the administration of antibodies from other species may theoretically cause IgE-mediated allergic reactions or Arthus' serum sickness, no adverse clinical events were reported for IgYs administered via the respiratory or gastrointestinal tract [6, 14]. And more clinical data is needed to assess the adverse reactions of IgYs. However, there is no doubt that IgYs have a bright future in the prevention and treatment of infectious diseases in humans [6, 8, 14, 15].

We used the inactivated whole *V. vulnificus* bacteria to immunize laying hens. Our present results show that the specific IgYs thus produced exert significant prophylactic and therapeutic effects on *V. vulnificus* infection. They could reduce the inflammatory response and blood bacterial load and keep at 100% the survival rate of *V. vulnificus*-infected mice. No obvious adverse reactions occurred in the IgY-treated mice. Therefore, our findings constitute the cornerstone of future clinical trials. Interestingly, the nonspecific IgYs also exerted partial inhibitory effects in vitro and mitigated the inflammatory response and lowered the blood bacterial load in vivo in comparison with the control (No IgYs) group. This might be ascribed to the fact that both the nonspecific and the specific antibodies recognized and bound portions of the same epitopes. However, aside their partial prophylactic and therapeutic effects on *V. vulnificus* infection, the nonspecific antibodies could not improve the survival rate of *V. vulnificus*-infected mice.

In the present study, inactivated whole *V. vulnificus* bacteria served as antigens for immunization. Earlier studies had shown that VvhA and RtxA1 were the main virulence factors of *V. vulnificus* infection as silencing the corresponding genes significantly reduced the virulence of *V. vulnificus* strains [12, 13, 16]. Therefore, we plan to use VvhA and RtxA1 recombinant proteins as antigens to produce specific IgYs and to explore the effectiveness of the latter in *V. vulnificus* infections.

## 5. Conclusions

Our study showed that specific IgYs showed an excellent prophylactic and therapeutic efficacy against *V. vulnificus* infections. These antibodies could significantly mitigate the inflammatory response and lower the blood bacterial load while keeping at 100% the survival rate of infected mice. These preclinical findings constitute the foundations for future clinical trials confirming the effectiveness of specific IgYs in preventing and treating *V. vulnificus* infections.

## Data Availability

The authors will supply the study data following reasonable requests.
